# Identification of the Specific Spoilage Organism in Farmed Sturgeon (*Acipenser baerii*) Fillets and Its Associated Quality and Flavour Change during Ice Storage

**DOI:** 10.3390/foods10092021

**Published:** 2021-08-28

**Authors:** Zhichao Zhang, Ruiyun Wu, Meng Gui, Zhijie Jiang, Pinglan Li

**Affiliations:** 1 Beijing Laboratory for Food Quality and Safety, College of Food Science and Nutritional Engineering, China Agricultural University, Beijing 100083, China; zhgio2008@126.com (Z.Z.); wry0814@cau.edu.cn (R.W.); 2 Jiangxi Institute of Food Inspection and Testing, Nanchang 330001, China; 3 Beijing Fisheries Research Institute, Beijing 100083, China; guimeng172@126.com; 4 NMPA Key Laboratory for Research and Evaluation of Generic Drugs, Beijing Institute for Drug Control, Beijing 102206, China; Jiangzhijie@126.com

**Keywords:** sturgeon, *Shewanella*, *Pseudomonas*, spoilage, flavour

## Abstract

Hybrid sturgeon, a popular commercial fish, plays important role in the aquaculture in China, while its spoilage during storage significantly limits the commercial value. In this study, the specific spoilage organisms (SSOs) from ice stored-sturgeon fillet were isolated and identified by analyzing their spoilage related on sensory change, microbial growth, and biochemical properties, including total volatile base nitrogen (TVBN), thiobarbituric acid reactive substances (TBARS), and proteolytic degradation. In addition, the effect of the SSOs on the change of volatile flavor compounds was evaluated by solid phase microextraction (SPME) and gas chromatography-mass spectrometry (GC-MS). The results showed that the *Pseudomonas fluorescens*, *Pseudomonas mandelii*, and *Shewanella putrefaciens* were the main SSOs in the ice stored-sturgeon fillet, and significantly affect the odors by changing the volatile compounds in the sturgeon. Compared with the fresh sturgeon, the appreciable increase of polycyclic aromatic hydrocarbons and tetramethyl-pyrazine might be the spoilage indicators of the sturgeon contaminated by *P. fluorescens*; the appreciable increase of 1-octen-3-ol and (z)-2-penten-1-o might be the potential marker of the sturgeon contaminated by *P. mandelii*; and the appreciable increase of 1-(3,3-dimethylbicyclo [2.2.1] hept-2-yl)-ethanon and butylated hydroxytoluene were associated with *S. putrefaciens*. This study reveals the relationship between the SSOs and flavor changes in sturgeon fillets, which will contribute to the sturgeon preservation and shelf-life extension.

## 1. Introduction

Sturgeon, one of the oldest fish in the world, is of great biological and economical importance. In 2020, the production of sturgeon in the world was ~102 million tons [1]. Sturgeon exhibits high economic and nutritional value due to its great utilization of body components, including its flesh, skin, caviar, and cartilage [2]. The content of essential amino acids and polyunsaturated fatty acids is rich in sturgeon flesh, especially the decosahexaenoic acid (DHA) and eicosapentaenoic acid (EPA) are ~12.5% which is more than in salmon. Sturgeon can be processed into raw fish fillets and smoked fish slices [3,4], which can be found in the current market. However, due to the high water content, rich nutrients, high endogenous enzyme activity, and near neutral pH value, sturgeon meat products are easy to get spoilage with deteriorated taste and flavor under the joint action of endogenous enzymes and microorganisms during the processing, transportation, and storage [5], which can cause health issue as well as reduce its commercial value. Generally, the initial deterioration of fresh fish quality is caused by autolysis, while subsequently by bacterial activity [6] and the microbial growth plays the major role in the fish spoilage. Fresh caught fish is naturally contaminated with various microbes, and only a small portion of microorganisms can survive afterwards. However, just the small portion of microorganisms cause fish spoilage during storage due to their great tolerance to the preservation conditions, and those microorganisms are named as “specific spoilage organisms (SSOs)” [7,8]. Recently, numerous works have accompanied microbiota analysis with the evaluation of quality changes to trace these correspondences [9,10,11]. However, different microorganisms show different spoilage characteristics, especially in fish. Biogenic amines, such as histamine, putrescine and cadaverine, are considered as indicators of fish spoilage [12]. Therefore, the current research mainly focuses on the analysis of biogenic amines content, but there is no significant difference. Meanwhile, at the beginning of spoilage, proteins, amino acids and other nitrogen-containing substances in the flesh are decomposed under the action of microorganisms. Then some soluble and small molecular compounds are degraded into volatile metabolites, such as ethanol, ketamine, sulfide, indole, aldehydes, esters and low-grade fatty acids [13,14], which cause the deterioration of the meat texture and color as well as produce the bad odors and toxic compounds [15,16]. In seafood products, the ammonia like odor and sour taste were the main problem to maintain the quality during storage. Research indicates that different dominant spoilage bacteria species may affect the fish microbial ecosystem differently, and therefore play different roles to affect the quality. *Shewanella putrefaciens* is a famous marine fish spoilage bacterium, producing trimethylamine and H_2_S in the process of metabolism, which brings strong odor [17,18]. *Photobacterium phosphoreum*, associated with acetic acid production, is considered as a spoilage marker in modified atmosphere packaged raw salmon [19]. Production of volatile compounds, such as trimethylamine, ethyl acetate and butanol were believed to play important role in *Serratia liqefaciens* spoiled cold-smoked salmon [18]. The *Carnobacterium maltaromaticum* can produce 3-methyl-1-butanal, 2-methyl-1-butanal, 2, 3-butanedione and ethyl acetate, which is also a major spoilage bacterium in fish products [19]. Other typical spoilage bacteria, such as *Pseudomonas fragi*, *Pseudomonas fluorescens,* and *Shewanella putrefaciens* also showed different metabolic characteristics when inoculated into sterile *Sebastes melanops* muscle [20,21,22,23,24]. In recent years, some literature has reported the relationship between dominant spoilage bacteria from fish and volatile compounds. However, the relationship between the SSOs of sturgeon and their effect on sturgeon products quality change is still missing, especially their role in affecting volatile flavor compounds. At the same time, because sturgeon has a high water content, neutral pH, and rich nutrition, it is very easy to cause microcirculation after death, biological proliferation leads to corruption. Under specific conditions, only part of the microorganisms contained in fish meat participate in the corruption process. Therefore, studying the specific spoilage bacteria and their putrescence ability of fish meat can clarify the corruption process and corruption mode of fish meat, and provide a research basis for the development of safe and efficient preservation technology, reliable shelf-life prediction technology, and freshness indicators.

In this study, the SSOs were isolated and identified from ice stored-aerobic tray packaging sturgeon fillets by analyzing their spoilage potential on sensory change, microbial growth, and biochemical properties, including total volatile base nitrogen (TVBN), thiobarbituric acid reactive substances (TBARS) and proteolytic degradation. Meanwhile, the effect of the SSOs on the change of volatile flavor compounds was evaluated by solid phase microextraction (SPME) and gas chromatography-mass spectrometry (GC-MS).

## 2. Materials and Methods

### 2.1. Isolation and Identification of Strains

The dominant spoilage bacteria in sturgeon fillets stored aerobically on ice were isolated and identified by a combination of culture-dependent and independent (PCR-DGGE) methods [25]. Meanwhile, different typical morphological colonies were randomly isolated from plates of Cetrimide Fucidin Cephaloridine (CFC, Hope Biol-Technology Co., Ltd., Qingdao, China) and Iron Agar (black colonies) (IA, Hope Biol-Technology Co., Ltd., Qingdao, China), respectively. Five *Pseudomonas* species (*Pseudomonas mandelii* (J-2, J-7, J-8, J-9), *Pseudomonas fragi* (J-5)), *Pseudomonas deceptionensis* (J-12), *Pseudomonas fluorescens* (J-1, J-11, J-13), and *Pseudomonas* sp. J-10) and 2 *Shewanella* species (*Shewanella putrefaciens* (X-1, X-2), *Shewanella baltica* (X-3)) were identified by 16S rRNA gene and rpoD gene. Spoilage characteristics of the seven different bacteria were analyzed in the study.

#### 2.1.1. Total Bacterial DNA Extraction and 16S rDNA V3 Variable Region Amplification

We aseptically weighed a certain meat sample, cut it with sterilized scissors, and then extracted DNA with bacterial genome rapid extraction kit (Beijing Biomed Technology Development Co., Ltd., Beijing, China).

Nested PCR was used to identify the isolated SSOs according to wang et al. [25] Briefly, the primer 8F: 5′-GGAGAGTTTGATCACTGGCT-3′ and 798R: 5′-CCAGGGTATCTAATCCTGTT -3′ were used to amplify about 750 bp of 16S rDNA sequence in the first PCR reaction; and the primer GC338F: 5′-CGCCCGCCGCGGGGCGGGGCACGGGGGGACTCCTACGGGAGGCAGCAG-3′, 518R: 5′-ATTACCGCGGCTGG-3′ were used to amplify the V3 region of 16S rDNA in the second PCR reaction.

#### 2.1.2. DGGE Analysis

Referring to Wang methods [25] with appropriate modification, DGGE analysis of target amplification products in bacterial 16S rDNA V3 region was carried out: polyacrylamide gel concentration was 10% (acrylamide: methylene bisacrylamide 37.5:1), denaturation gradient: 30–60% (100% denaturant containing 7 mol/L urea and 40% deionized formamide). Electrophoresis conditions: at 0.5 × TAE buffer, at 80 V for 5 h, then at 60 V for 16 h at a constant temperature of 60 °C. After electrophoresis, the gel was stained with GelRed nucleic acid dye for 20 min.

After that, under UV irradiation, DGGE strips at different positions were cut off with a sterile blade and put into 1.5 mL EP tube, adding about 40 μL ddH_2_O, and 4 °C overnight. Primers 338F: 5′-ACGGGAGGCAGCAG-3′, 518R: 5′-ATTACCGCGGCTGG-3′ were used for amplification. The products were purified and sequenced by Biomed Company (Beijing, China). The results were compared and analyzed in NCBI.

### 2.2. Sterile Fillet Sample Preparation

A total of 15 commercially farmed sturgeon (*Acipenser baerii*) were used in this experiment. Each fish weighed about 7–8 kg and was about 4 years old, was purchased from Sturgeon Farm of Beijing Fisheries Research Institute (Beijing, China). Sterile sturgeon fillets (about 30 g each fillet) were prepared according to the method described by Huang [26]. After the purchased fresh sturgeon were knocked to death, they were immediately cleaned with 50 g/L Na_2_CO_3_ solution to remove the surface mucus and the head, tail, and internal organs. Under the sterile environment, fish were washed 50 g/L Na_2_CO_3_ solution and 2% formalin solution in turn, cut into fish slices (about 30 g per piece) under the sterile condition, washed thoroughly with sterile water and drained. After culturing and counting, the total number of bacteria in sterilized fish fillets was less than 2 Log (CFU/g). After being made into sterile fillets, the fillets were immediately placed in a sterile container and placed in a super clean bench. Then they were divided randomly into four groups.

All *Pseudomonas* and *Shewanella* isolates were grown individually in Nutrient Broth (NB, Hope Biol-Technology Co., Ltd., Qingdao, China) at 30 °C for 48 h. The inoculation mixture was diluted to approximately 5 Log (CFU g^−1^) with sterile physiological saline. Sterile sturgeon fillets were soaked in the inoculation mixture for 15 s, and drained. The initial inoculated concentration was 3–4 Log (CFU g^−1^). The control was performed by inoculating sterile physiological saline into sterile sturgeon fillets. The inoculated fillets were wrapped with a thin polyethylene film and placed in a polystyrene box, then covered with a brittle layer of ice which was replaced every day to maintain a steady temperature. The samples were stored refrigerated at 4 ± 0.5 °C for 18 days and analyzed on days 0, 3, 6, 9, 12, 15, and 18.

### 2.3. Sensory Analysis

The sensory was analyzed according to the Quality Index Method (QIM) described by Hovda [27] with some modifications. The sensory panel consists of six trained evaluators. The panelists were required to score the color, brightness, transparency, odor, surface mucus, and texture. The scores were 0–3, wherein 0 denoted the best quality and 3 denoted the worst quality. The QIM value was the sum of the scores on different quality parameters given by each panelist.

### 2.4. Microbiological Analysis

Minced sturgeon flesh (10 g) was aseptically transferred into a polyethylene tube containing 90 mL sterile peptone water (0.85% NaCl, 0.1% peptone) and homogenized for 2 min. Serial 10-fold dilutions of the homogenate were carried out in sterile physiological saline. Poured-plate method was used for colony count. The total viable count was incubated on Plate Count Agar (PCA, Hope Biol-Technology Co., Ltd., Qingdao, China) at 30 °C for 3 days.

### 2.5. Biochemical Analysis

The pH was measured in 10-fold diluted flesh with a digital pH meter (PB-10, Satroris, Beijing, China) following the current Determination of pH value of Chinese national food safety standard (GB 5009.237-2016). Briefly, 1 g sturgeon meat was added in 5 mL normal saline. After homogenization, the pH of the resulting homogenate was determined using a pH meter. Total volatile basic nitrogen (TVBN) was measured following the current Chinese national food safety standard (GB 5009.228-2016). Thiobarbituric Acid Reactive Substances (TBARS) was measured following the method described by Botsoglou et al. [28] TCA-soluble peptide was measured following the method described by Broekaert et al. [29].

### 2.6. Determination of Histamine

The determination of histamine during storage was carried out according to the method provided in the instructions of the kit (308-16121, China Baobai Biotechnology Co., Ltd.). The mechanism is that histamine in the action of histamine dehydrogenase occurs with a color reaction. According to the protocol supplied instructions, we prepared the histamine in standard solution, mixed and diluted to 50, 25, 20, 10, 5, 2.5, 2, and 1 μg/mL, used to draw the marked curve. Firstly, the fish meat was stirred thoroughly and 24 mL of 0.1 M EDTA were added. The mixture was heated in a water bath for 20 min. Then the supernatant was obtained by filtration with qualitative filter paper (Hangzhou Special Paper Co., Ltd., Hangzhou, China) and the supernatant was treated with chromogenic agent (1 mL) and histamine dehydrogenase solution (0.5 mL). After being kept away from light for 15 min at 37 °C, the absorbance was measured at 470 nm, and the corresponding histamine content was calculated using the standard curve (Y = 0.0096X − 0.0042, R^2^ = 0.9972, Y represents the histamine concentration (μg/mL) in the sample, and X is the absorbance value at 470 nm). 

The proteins of sturgeon flesh were stored for 0 and 18 days, and were extracted according to the method described by Li et al. [30] with some modifications. Sodium dodecyl sulfate-polyacrylamide gel electrophoresis (SDS-PAGE) (Bio-Rad, Hercules, CA, USA) was performed using 10% separating gel with 5% stacking gel. The supernatant of total-soluble protein (10 μL), water-soluble protein (16 μL), and salt-soluble protein (12 μL) was loaded onto the gradient polyacrylamide gel made of 5% stacking gel and 10% separating gel. Then electrophoresis was performed at a voltage of 80 V for 20 min, followed by a voltage of 120 V for 40 min. After separation, gels were stabilized in fixative (50% ethanol and 10% acetic acid) for 60 min and subsequently immersed in stainer (10% acetic acid, 50% ethanol, 0.25% Coomassie brilliant blue R-250) for 60 min and destainer (25% ethanol, 8% acetic acid) for 90 min.

### 2.7. Volatile Compounds Analysis

The volatile compounds produced by spoilage sturgeon fillets inoculated with *Pseudomonas fluorescens*, *Pseudomonas mandelii*, and *Shewanella putrefaciens*, were analyzed. The non-inoculated sterile sturgeon fillets were prepared as control. The vial system was equilibrated at 60 °C for 20 min. The solid-phase micro-extraction (SPME) fiber (65 μm PDMS/DVB, Supelco, Bellefonte, PA, USA) was used to absorb volatile compounds at 60 °C for 30 min, then inserted into the injector port of gas chromatograph in the splitless mode for 2 min at 250 °C. Volatile compounds were determined by gas chromatograph coupled with mass spectrometry (GC-MS, QP2010 plus, Shimazu, Japan). The oven temperature was held at 40 °C for 30 min, increased to 120 °C at the speed of 5 °C min^−1^, then ramped at 10 °C min^−1^ to 230 °C, and held for 5 min. The scan of masses ranged from 35 to 500 *m*/*z*. The NIST11 library and comparison of mass spectra and linear retention indexes with those of standards injected in the same conditions were used to identify the volatile compounds. The relative content of volatile compounds was determined with area normalization method. The GC conditions were as follows: the initial temperature of the column incubator was 40 °C, the temperature of the injection port was 250 °C, no split injection, the flow rate of carrier gas (He) was 1 mL/min, the temperature rising program of the column incubator was 40 °C for 30 min, 5 °C/min for 120 °C, 10 °C/min for 230 °C for 5 min. MS conditions: the temperature of ion source was 200 °C, the temperature of transmission line was 250 °C, the signal was collected in full scan mode, and the scanning range was 35–500 *m*/*z*.

### 2.8. Statistical Analysis

All experiments were conducted in triplicate and results were expressed as mean ± standard deviation. Principal component analysis (PCA) was employed to visualize differences in multiple parameters on the average data of samples among groups. Comparisons of multiple groups were analyzed by one-way analysis of variance (ANOVA) with Duncan’s multiple range test using SPSS 22.0 (SPSS Inc., Chicago, IL, USA) software (significance was defined at *p* < 0.05).

## 3. Results and Discussion

### 3.1. Bacterial Strains

**Figure 1:**
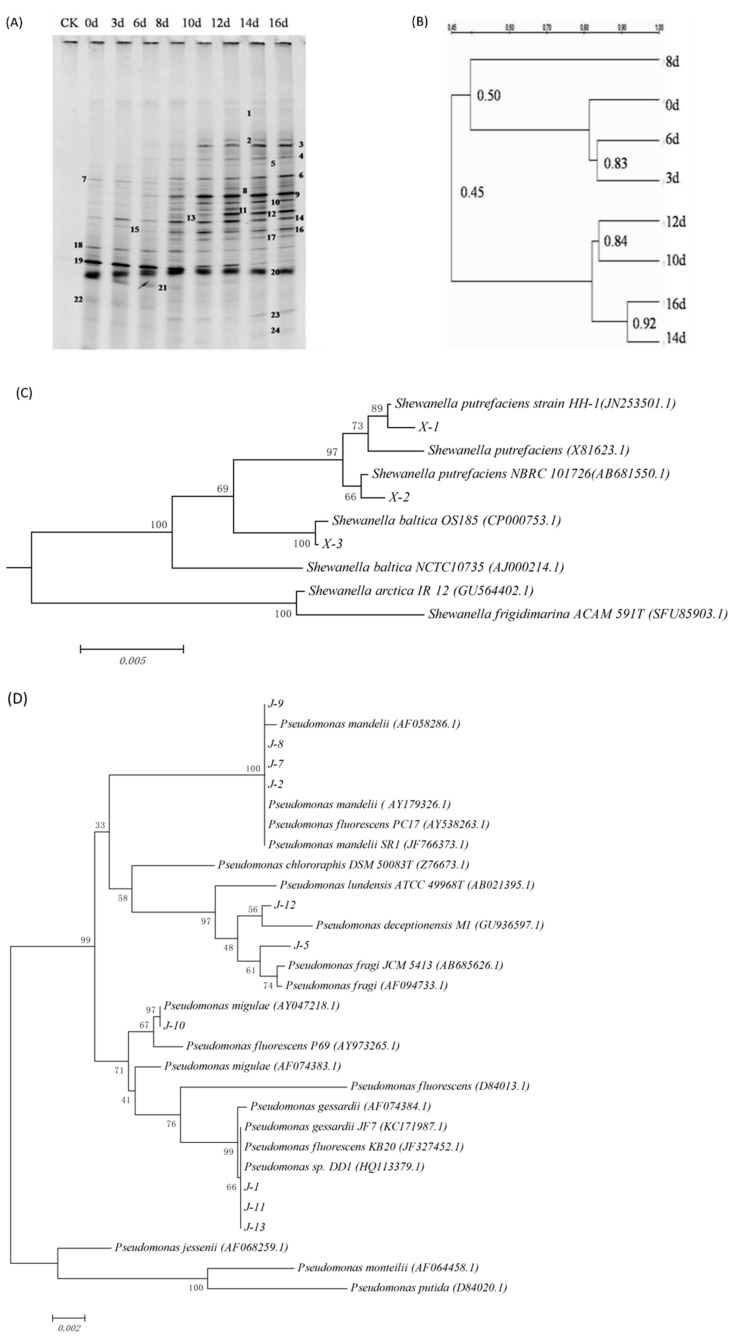
Analysis of DGGE Atlas. DGGE profile of bacteria from sturgeon stored aerobically on ice (**A**). Cluster analysis of DGGE bacteria profile (**B**). Phylogenetic tree of the 16S rRNA gene of *Shewanella* sp. isolated from sturgeon fillets (**C**). Phylogenetic tree of the 16S rRNA gene of *Pseudomnas* sp. isolated from sturgeon fillets (**D**).

In general, the bands at different positions in the DGGE map represent different microbial species, and brighter bands indicates greater number of individual bacteria. It can be seen from Figure 1A that during the ice storage process, with the increase of storage time, more bands can be detected by DGGE, and the brightness of the bands at the same position changed, which indicates that during the storage process, the microbial community structure changed dynamically over time. The dominant bacterial species during different storage periods is different.

In order to further clarify the relationship of microbial community structure in fish meat with different storage time, an unweighted pair group method with arithmetical means (UPGMA) method was used to construct the DGGE map. The results are as shown in Figure 1B: according to the similarity of bacterial communities, the flora at 0, 3, 6, and 8 days are grouped into one cluster, and that on day 10, 12, 14, and 16 are in a separate cluster. During the storage process, microorganisms need a period of adaptation to the new environment, so the flora changes little during the early stage of storage, and the similarity is greater than 80% among the samples collected at 0, 3, and 6 days; with the increase of storage time, the microorganisms in fish continue to grow and the dominant spoilage bacteria, which causes the fluctuation of the microbial community structure, among which the flora on the eighth day and days 0, 3, and 6 is more than 80%, the similarity was only 50%, but in the subsequent storage stage, the bacterial community structure gradually tended to be stable, and the similarity between the 10th day and the 12th day was 84%, and the similarity between the 14th day and the 16th day was as high as 92%.

The results of bacteria identification are shown in Table 1. According to DGGE patterns (Figure 1A) and 16S rRNA sequencing analysis (Figure 1C,D), the dominant bacteria in fresh sturgeon were *Pseudomonas* sp. (bands 7, 14, 18), *Shewanella* sp. (bands 20), *Acinetobacter* sp. (bands 19) and *Escherichia* sp. (bands 22). With the increase of storage time, the brightness of bands 3, 4, 6, 9, 10, 12, and 16 increased, indicating that the dominant position of *Acinetobacter* sp. in microbial flora gradually lost, while the *Shewanella* sp., *Pseudomonas* sp., and other microorganisms gradually became dominant bacteria during the late storage period [31]. *Shewanella* sp. and *Pseudomonas* sp. are psychrophilic bacteria and can grow at 0 °C. *Pseudomonas* sp. grows rapidly under aerobic conditions and has strong protein decomposition ability; *Shewanella* sp. can reduce trimethylamine oxide, producing hydrogen sulfide that can react with pigment in meat tissue, resulting in fish discoloration and produced rancid smell [32].

### 3.2. Microbiological Analysis

**Figure 2:**
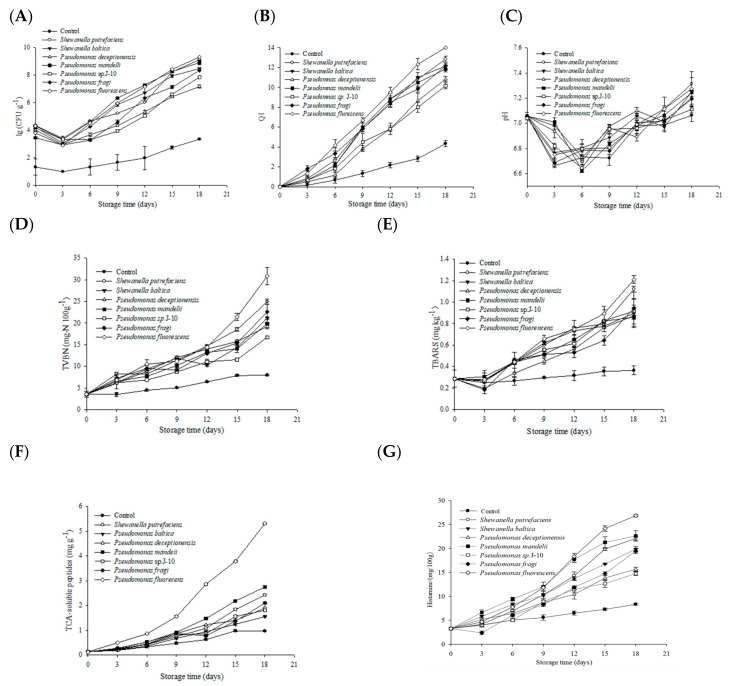
Growth of total bacteria (**A**), sensory (**B**), pH (**C**), total volatile base nitrogen (TVBN) production (**D**), thiobarbituric acid reactive substances (TBARS) (**E**), TCA-soluble peptides (**F**), and histamine (**G**) analysis of sturgeon fillets inoculated with different bacterial groups stored aerobically on ice.

The results of the total viable counts of different bacteria groups are shown in Figure 2A. The initial count of the control was 1.33 ± 0.47 Log (CFU g^−1^), and increased to 3.35 ± 0.05 Log (CFU g^−1^) at the end of storage, which showed significant lower values than that of inoculated groups. The initial counts of different inoculated samples were approximately 4 Log (CFU g^−1^) as expected. *Pseudomonas fluorescens* and *Pseudomonas mandelii* grew fast after a decline within the initial 3 days. The count was 7.15 ± 0.01 and 7.29 ± 0.05 Log (CFU g^−1^), respectively, at day 12. Meanwhile, *Shewanella putrefaciens* began to grow rapidly after the sample stored for 12 days. The count reach 8.24 ± 0.03 Log (CFU g^−1^), and showed no significant difference from that of *Pseudomonas fluorescens* group and *Pseudomonas mandelii* group at the end of storage (*p* < 0.05). The other three bacteria grew at a slow rate, and showed a significant difference compared to others at the later period of storage. Generally speaking, the microorganisms contained in fish from freshwater waters mainly included *Pseudomonas* and a wide variety of G^+^ bacteria. Reynisson et al. [33] studied the flora structure of North Atlantic cod under the conditions of 0 °C and micro freezing storage (−2 and −3.6 °C). The results showed that *Pseudomonas* was the dominant bacteria for fish meat corruption after storage at 0 °C. Li et al. [34] also made a similar study and compared the bacterial composition of fresh carp slices and thawed carp slices after 4 weeks of freezing at 4 °C. It was found that the dominant bacteria in the spoilage of the two groups were *Pseudomonas*. It is consistent with our results.

### 3.3. Sensory Analysis

Sensory characteristics of different inoculated sturgeon fillets stored aerobically on ice were analyzed. As is shown in Figure 2B, the control was not considered to be spoiled throughout the 18-day incubation period. *Pseudomonas fluorescens* inoculated group and *Pseudomonas mandelii* inoculated group reached the end of shelf life at day 12, and the corresponding QI values were: 9.50 ± 0.50 and 8.83 ± 0.58, respectively. The samples of *Shewanella putrefaciens* inoculated group, *Shewanella baltica* inoculated group, and *Pseudomonas fragi* inoculated group reached sensory rejection point after 15 days. However, samples of the two other bacterial groups began to deteriorate at day 18. Similar studies have found that modified atmosphere packaged dehydrated pickled cod (Gadidae) can produce dark brown mucus under the action of microorganisms [35], while the meat of sterile bighead carp back connected to *Pseudomonas* sp. and *Shewanella* sp. is light green and light yellow respectively at the later stage of refrigeration [36]. Similar to our results, it shows that microorganisms are the main factor causing fish meat discoloration.

### 3.4. Biochemical Analysis

The initial pH value of sterile sturgeon fillets was 7.06 ± 0.03 (Figure 2C). Overall, all samples showed a trend of pH increase after a slight decreasing at day 6. This is mainly because after the death of live fish, ATP, phosphocreatine and other substances decompose to produce acidic substances such as phosphoric acid. At the same time, glycogen metabolism produces lactic acid, which reduces the pH in fish meat. However, with the increase of storage time, endogenous enzymes and surface microorganisms decompose nitrogen-containing compounds such as protein, producing more alkaline substances, resulting in the increase of pH [31,37]. However, the pH of each experimental group basically fluctuated due to the low ice storage temperature, the slow decline of fish freshness, the insufficient production of acidic substances metabolized by the body, and the accumulation of alkaline substances. So, the of pH value of *Pseudomonas fluorescens* group and *Shewanella putrefaciens* group was higher than that of other bacteria groups at the end of storage, which was 7.35 ± 0.02 and 7.31 ± 0.05, respectively. Similar studies found that the pH of grass carp fillets also decreased first and then increased at −3 °C and 0 °C [31], which is consistent with our research results.

Although the TVBN production of different bacteria groups increased slowly at the early period of storage, it was significantly higher than that of the control during storage (Figure 2D). *Pseudomonas fluorescens* group, *Shewanella putrefaciens* group, and *Shewanella baltica* group produced a large amount of TVBN rapidly after being stored for 12 days. Meanwhile, *Pseudomonas fluorescens* group had the maximum production rate of TVBN, which showed a significant difference compared to other groups, and reached 30.80 ± 1.98 mg-N 100 g^−1^ at the end of storage. The TVBN value of the four other bacterial groups also increased dramatically after being stored for 15 days.

The TBARS value has been widely used to describe the degree of lipid oxidation. As is shown in Figure 2E, the TBARS value of the control did not change significantly as the storage time extended. Meanwhile, TBARS values of *Pseudomonas fluorescens* group, and *Shewanella putrefaciens* group were higher than other bacteria groups at the end of storage, reaching 1.21 ± 0.03 and 1.12 ± 0.03 mg MDA kg^−1^, respectively. The TBARS value of *Pseudomonas mandelii* group was 0.86 ± 0.02 mg MDA kg^−1^, which was the minimum value among all bacteria groups at day18.

Changes of TCA-soluble peptides of different bacteria groups are shown in Figure 2F. The TCA-soluble peptides production of all bacteria groups increased with storage time extended. The TCA-soluble peptides content of *Pseudomonas fluorescens* group and *Pseudomonas mandelii* group increased rapidly after being stored for 9 days, and the similar phenomenon was also found in *Shewanella putrefaciens* group and *Pseudomonas fragi* group. *Pseudomonas fluorescens* inoculated group showed the highest amount of TCA-soluble peptides.

Spoilage characteristics of *Pseudomonas mandelii* were rarely studied. The samples inoculated with *Pseudomonas mandelii* also spoiled very quickly and the proteolytic activity was stronger than other inoculated bacteria except *Pseudomonas fluorescens*, though its TVBN and TBARS values were not high. *Pseudomonas fragi* has also been identified as the dominating species in meat [38]. Although sensory, microbiological, and some biochemical indexes increased fast in the end of storage in *Pseudomonas fragi* inoculated samples, these values indicated that the spoilage potential of *Pseudomonas fragi* did not stand out when compared with other inoculated species in this study. *Pseudomonas deceptionensis* and *Pseudomonas* sp. J-10 was found to have the weakest spoilage potential on sturgeon fillets: the dominance of *Pseudomonas* species depends partly on its ability to metabolize glucose, creatine, and creatinine [39]. Therefore, *Pseudomonas* species such as *Pseudomonas fluorescens* that can utilize proteins as its major nutrient sources to grow fast will likely become the dominant species. However, the proteolytic activity of *Shewanella putrefaciens* was not prominent, but it grew fast. It is possible that *Shewanella putrefaciens* feed on the proteolytic molecules produced by other bacterial species. As for *Shewanella baltica*, biochemical analysis has shown that the spoilage potential was moderate, though it grew at a fast rate as well as *Shewanella putrefaciens*. It was found that *Shewanella* species could produce off-odor compounds, such as H_2_S, which can react with muscle pigments, and result in a green discoloration [40].

### 3.5. Changes of Histamine

The changes of histamine content of the tray packed sturgeon inoculated with different strains during ice storage are shown in Figure 2G the histamine content of the fish in each experimental group showed an upward trend during storage. In the early stage of storage, histamine production was less. On the 9th day, the histamine content of fish in each treatment group was significantly different from that in the blank control group. This was mainly due to the small number of microorganisms, less histidine decarboxylase production and slow change of *histamine* content. However, with the gradual adaptation of microorganisms to the storage environment, microbial growth and metabolism were vigorous. The increase of histamine content in each experimental group was accelerated. After 9 days of storage, the histamine content of *Pseudomonas fluorescens* group and *Pseudomonas mandelii* group increased significantly. On the 15th day, the histamine content of *Pseudomonas fluorescens* group was significantly different from other experimental groups, reaching 24.170 ± 58 mg/100 g, while the histamine content in *Pseudomonas deceptionensis* group and *Pseudomonas* sp. J-10 group increased slowly during storage, and reached 15.650 ± 0.41 mg/100 g, 14.76 ± 0.39 mg/100 g. This indicated that the ability of different microorganisms to promote histamine production in fish was different, among which *Pseudomonas fluorescens* was the strongest and *Pseudomonas* sp. J-10 was the weakest.

During the storage of fish, the free histidine is decomposed by histidine decarboxylase to produce histamine under the joint action of endogenous enzymes and microorganisms. Among many biogenic amines, histamine has the greatest impact on human health. Histamine poisoning caused by eating aquatic products occurs from time to time, so it has become an important index to evaluate the freshness of aquatic products [17]. Both *Pseudomonas* sp. and *Shewanella* sp. inoculated in this experiment were psychrophilic bacteria, and the activity of histidine decarboxylase was also inhibited due to the low temperature of ice storage, so the histamine content of each experimental group was still lower than the specified limit even after complete spoilage. However, the overall results still reflected the difference of histamine content among the experimental groups.

### 3.6. Proteolytic Degradation Analyzed by SDS-PAGE

**Figure 3:**
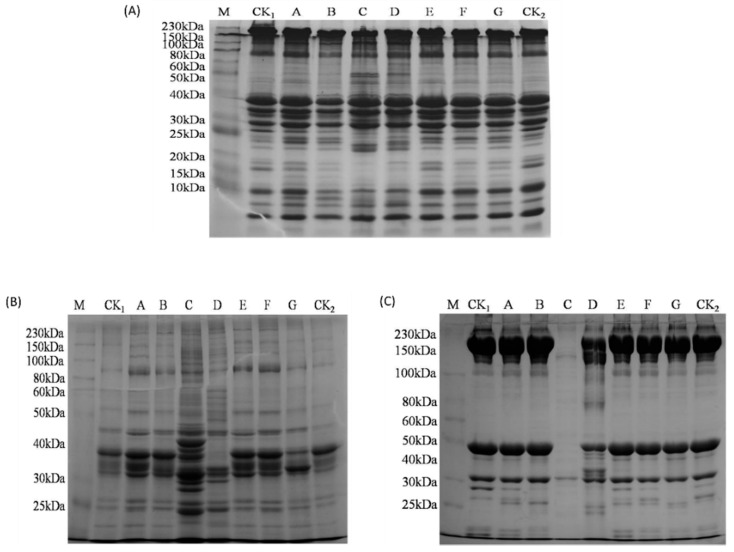
SDS-PAGE analysis of total soluble protein (**A**), water soluble protein (**B**), and salt soluble protein (**C**) of sturgeon fillets inoculated with different bacterial groups and stored aerobically on ice for 18 d. M, protein marker; CK1, control (stored for 0 d); A, *Shewanella putrefaciens*; B, *Shewanella baltica*; C, *Pseudomonas fluorescens*; D, *Pseudomonas mandelii*; E, *Pseudomonas* sp. J-10; F, *Pseudomonas fragi*; G, *Pseudomonas deceptionensis*; CK2, control (stored for 18 d).

The proteolytic degradation of different bacterial groups after being stored for 18 days was analyzed by SDS-PAGE. Twenty to thirty visual bands of total soluble protein were shown in Figure 3A, and the molecular weight ranged from 0 kDa to 230 kDa. No significant difference was observed among some main proteins of different bacteria inoculated groups such as myosin heavy chain (MHC, 230 kDa), α-actinin (100 kDa), actin (43 kDa), and tropomyosin (35 kDa). However, there were still different characteristics of the total soluble proteins among the different bacteria inoculated groups. The intensity of the bands with molecular weight of approximately 33, 25, 17, 15, and 10 kDa decreased *Shewanella baltica* group, *Pseudomonas fluorescens* group, and *Pseudomonas mandelii* group. The proteins of 15 kDa and 25 kDa were also degraded by *Shewanella putrefaciens*, *Pseudomonas deceptionensis*, and *Pseudomonas fragi*. Meanwhile, the intensity of the bands with molecular weight of approximately 22 kDa and 50 kDa in *Pseudomonas fluorescens* and *Pseudomonas mandeii* groups were higher than that of other bacteria groups. This may be the intermediate or oligomer of small protein molecules formed after protein degradation. Interestingly, compared with the total soluble proteins of other groups, a new protein band with the molecular weight less than 10 kDa was observed only in *Shewanella* species inoculated groups.

Changes of water-soluble proteins and salt-soluble proteins of different bacterial groups are shown in Figure 3B and Figure 3C, respectively. The bands of water-soluble proteins had the largest increase while salt-soluble proteins with similar molecular weight almost disappeared in the *Pseudomonas fluorescens* group. Meanwhile, as is shown in Figure 3B, the intensity of bands with the molecular weight of approximately 90 kDa became higher in different bacterial groups when compared with the control; the intensity of bands in approximately 40 kDa had a slight decrease in *Pseudomonas fluorescens* group and *Pseudomonas deceptionensis* group, while the proteins with similar molecular weight disappeared in *Pseudomonas mandelli* group.

MHC and actin degradation by *Pseudomonas mandelli* was observed in Figure 3C. An increase of intensity in bands with the molecular weight of approximately 25–40 kDa and 80–100 kDa was found in *Pseudomonas mandelli* group. As for other bacteria inoculated groups, proteins with molecular weight of 25 kDa to 30 kDa showed difference.

### 3.7. Effects of Specific Spoilage Bacteria on Volatile Flavor Compounds of Sturgeon

#### 3.7.1. Volatile Compounds Analysis

**Figure 4:**
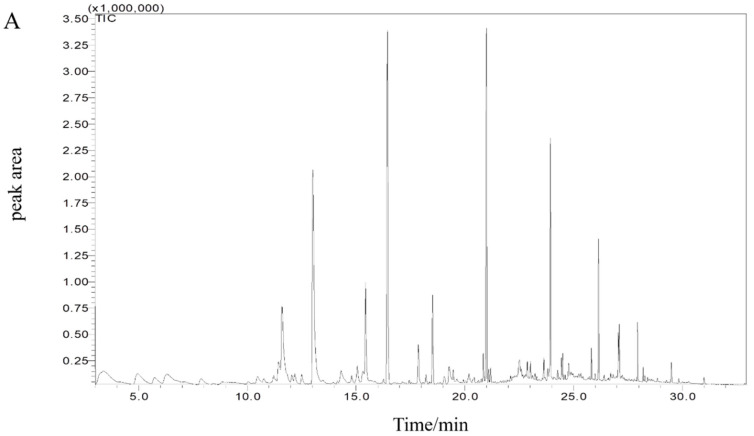

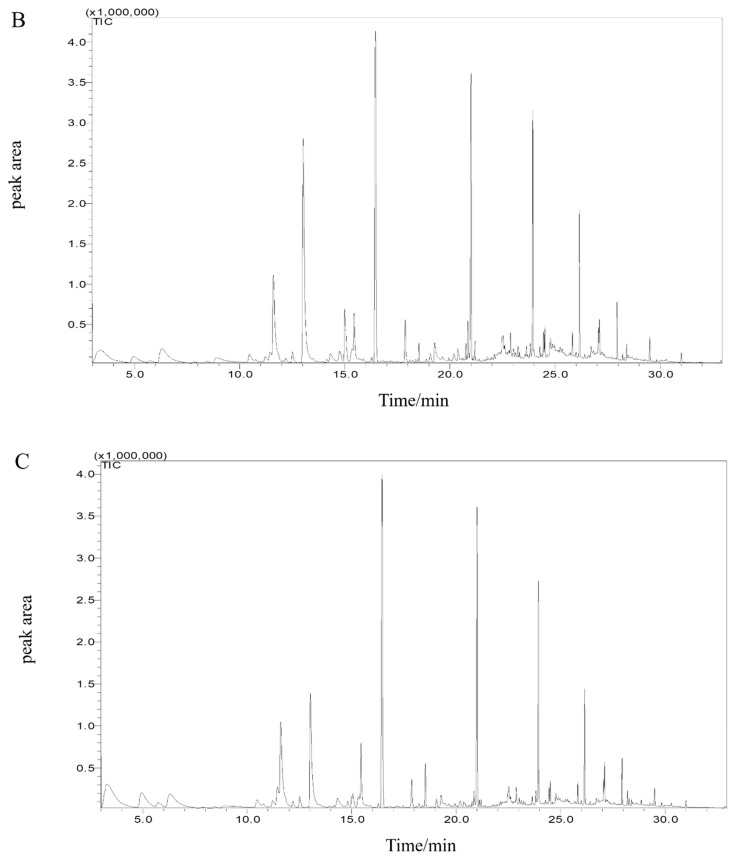

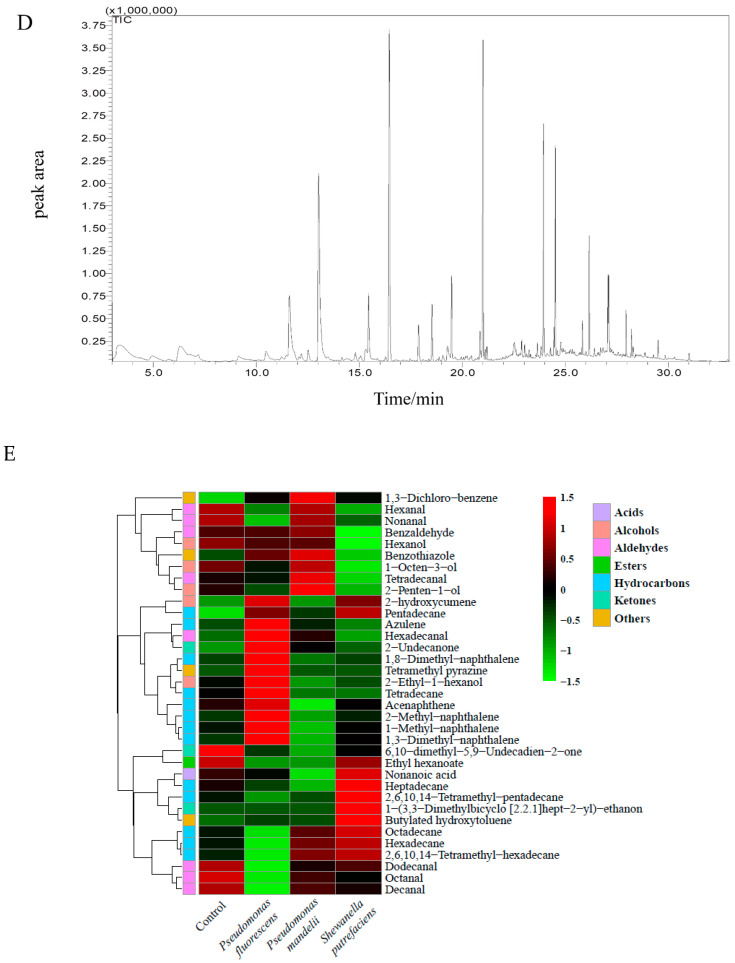
Total ion current diagram of volatile compounds and heat-map in sturgeon fillets inoculated with different bacteria stored aerobically on ice. Control (**A**); *Pseudomonas fluorescens* (**B**); *Pseudomonas mandelii* (**C**); *Shewanella putrefaciens* (**D**); heatmap (**E**). The level 3 pathways annotations were described on the left and colored according to level 2 categories. Acids, alcohols, aldehydes, esters, hydrocarbons, ketones, and other families were colored in violet, red, pink, green, blue, pistachio green, and yellow respectively. The hierarchical clustering tree was on left based on samples.

*Pseudomonas fluorescens*, *Pseudomonas mandelii*, and *Shewanella putrefaciens* were considered as strong spoilers based on the basic characteristics of spoilage potential. Volatile compounds produced by these species were further analyzed to find out the possible off-odor contributors. The aldehydes likely come from polyunsaturated fatty acid or amino acid catabolism, and considered as the typical flavor compounds in fresh fish [41]. Volatile compounds were used to analyze the characteristics of spoilage potential of those three bacterial species. Changes of volatile compounds of spoiled samples stored for 15 days are shown in Table S1. The GC-MS spectra of different inoculated samples were determined as shown in the Figure 4A–D, according to the peak time and retention time, 35 compounds with the highest content were determined, which included 8 aldehydes, 3 ketones, 1 acid, 1 ester, 5 alcohols, 13 hydrocarbons, and 4 other organic compounds. Changes of volatile compounds of spoiled samples stored for 15 days and peak numbers corresponded to volatiles compounds are shown in the Table S1. The relative contents of aldehydes, alcohols, and hydrocarbons varied greatly among different inoculated samples. To better visualize, a heat-map of the 35 compounds was constructed for different samples at different periods (Figure 4E). Each row represents a separate volatile substance, and each column represents the group used for analysis. In addition, different colors indicated the relative abundance of volatile species, in which high abundance were depicted in red, and conversely, low-abundant were marked in green.

The results show that in all fish samples, hexanal, octanal, nonanal, decanal, dodecanal, hexanol, and ethyl hexanoate content decreased. A corresponding decrease of most of the aldehydes was also found in all spoiled samples in this study when compared with the control. An increasing trend of 2-undecanone, 6, 10-dimethylundeca-5, 9-undecadien-2-one, and 1, 3-dichloro-benzene was shown in the spoiled samples, which implied that these compounds might be responsible for off-odors in spoiled sturgeon fillets. Some polycyclic aromatic hydrocarbons and tetramethyl-pyrazine might be the spoilage indicators of the samples inoculated with *Pseudomonas fluorescens*, polycyclic aromatic hydrocarbons were the off-odor contributors in fish previously. Pyrazine compounds were considered as the empyreumatic-odors source, and were possible to be produced by the condensation reactions between amino acid (ammonia) and dicarbonyl compounds or Maillard reaction. 1-(3, 3-dimethylbicyclo [2.2.1] hept-2-yl)-ethanon and ethyl hexanoate were the specific volatile compounds produced in *Shewanella putrefaciens* inoculated group, which might be the spoilage markers of this species. An increase of antioxidant BHT was also observed in *Shewanella putrefaciens* inoculated group. This compound was also found in spoiled salmon inoculated with Photobacterium phosphoreum [42]. Miller et al. [24] found that sterile Sebastes melanops inoculated with *Pseudomonas fluorescens* or *Shewanella putrefaciens* produced several sulfur-containing compounds (methyl mercaptan and dimethyl disulfide) and trimetylamine which were not detected in this study. This is likely due to the difference in the type of fish fillets and different experimental procedures. 1-octen-3-ol and (z)-2-penten-1-o increased significantly in *Pseudomonas mandelii* inoculated group, and this was different from other bacteria inoculated groups. This result implied that the spoilage characteristics of *Pseudomonas mandelii* might relate to unsaturated alcohols, which were considered to originate from oxidation of unsaturated fatty acids [43,44]. Similarly, previous studies used 1-octen-3-ol and (z)-2-penten-1-o as the potential markers for spoilage of other aquatic products [45,46].

#### 3.7.2. Principal Component Analysis (PCA)

**Figure 5:**
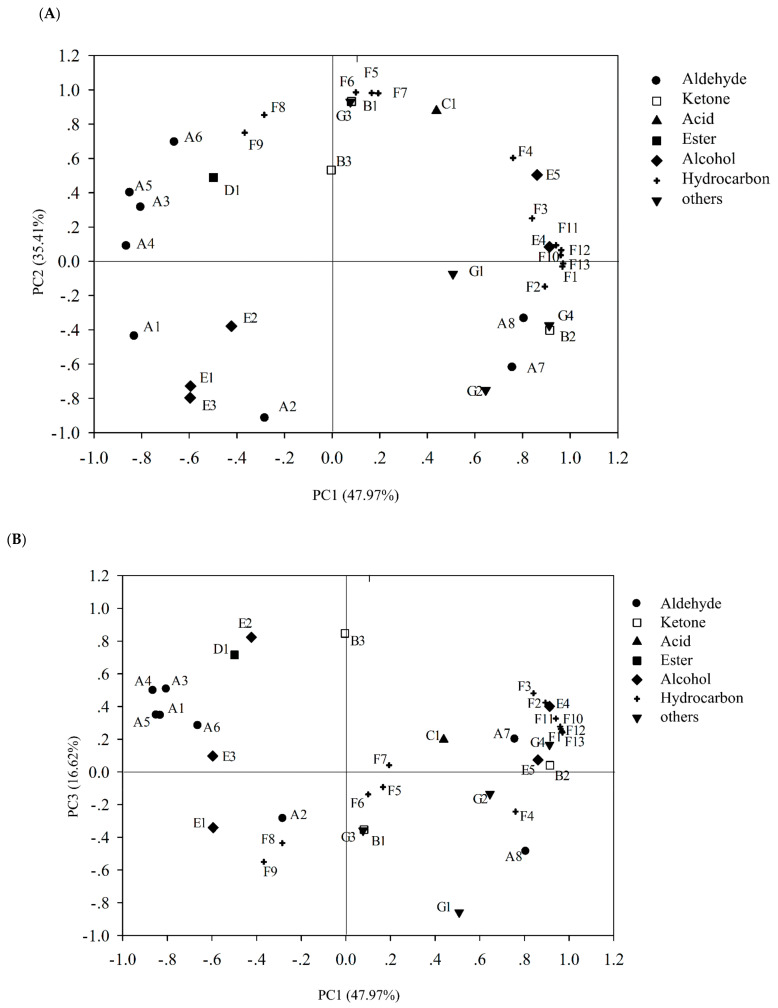
Variable loadings on plane1-2 (**A**) and 1-3 (**B**) of principle component analysis (PCA) on volatile compounds.

It should be noted that there may be some correlation between the changes of 35 volatile compounds detected. A certain overlap in the reflection of the odor characteristics of these volatile compounds should be considered. Therefore, PCA analysis was carried out to discover the correlation among aldehyde, ketone, acid, and other volatile compounds. The results showed three principal components were extracted, which could explain 47.98%, 35.41%, and 16.62% of the total information respectively (Figure 5). The PC1 was mainly negatively correlated with most aldehydes (hexanal, octanal, nonanal, decanal); it was negatively correlated with hexadecaldehyde, 1-[3,3-dimethylbicyclo [2.2.1] heptyl-2-yl] ethanone, octanol, 2-phenylisopropanol, osiridine, tetradecane, acenaphthene, tetramethylpyrazine, and all polycyclic aromatic hydrocarbons, octyl alcohol, 2-phenylisopropanol, osilane, and tetradecane. The second principal component mainly reacted with benzaldehyde, 1-[3,3-dimethylbicyclo [2.2.1] hept-2-yl] ethanone, nonanoic acid, 1-octen-3-ol, hexadecane, 2,6,10,14-tetramethylpentadecane, heptadecane, and di-tert-butyl-p-cresol. It can be seen from Figure 5B that the PC3 (16.62%) mainly explains the changes of 1,3-Dichlorobenzene; 6,10-dimethyl-5,9-undecane-2-one, and hexanol. These substances may be the main contributors to the odor changes of sturgeon during storage.

On this basis, the principal component scores of each experimental group were calculated (Table 2). The results showed that in the *Pseudomonas fluorescens* group, the greatest contribution comes from PC1, which indicated that some related volatile compounds might be the main source of odor of spoilage fish treated by *Pseudomonas fluorescens*. The PC2 had the greatest effect on the putrefaciens of *Shewanella putrefaciens* group, and only changed significantly in the fish treated by *Shewanella putrefaciens*, which may be related to the putrefaction characteristics of *Shewanella putrefaciens*.

## 4. Conclusions

*Pseudomonas fluorescens*, *Pseudomonas mandelii*, and *Shewanella putrefaciens* were strong spoilers on sturgeon fillets stored aerobically, by increasing TVBN content, fat oxidation degree and histamine content to stimulate fish spoilage. Different off-flavor characteristics of SSOs were performed. *Pseudomonas fluorescens* was correlated with the production of several polycyclic aromatic hydrocarbons and tetramethyl-pyrazine; 1-octen-3-ol and (z)-2-penten-1-o can be proposed as the spoilage marker of sturgeon spoiled by *Pseudomonas mandelii*; 1-(3,3-dimethylbicyclo [2.2.1] hept-2-yl)-ethanon and ethyl hexanoate were the specific volatile compounds produced in sturgeon inoculated with *Shewanella putrefaciens* and can be used as a spoilage marker for *Shewanella putrefaciensthus*.

## Figures and Tables

**Table 1:** 16S rDNA sequence similarities to closest relatives of DNA recovered from DGGE banks.

Number	Most Similar Strain	Login Number	Similarity
1	*Shewanella* sp. B157	FN295752.1	95%
2	*Pseudomonas* sp. 01WB03.2-36	FM161452.1	96%
3	*Uncultured bacterium clone* C16	AM411553.1	95%
4	*Shewanella baltica strain* SS3	JX032783.1	96%
5	*Shewanella baltica strain* ST5	JX032790.1	97%
6	*Shewanella baltica* OS678	CP002383.1	97%
7	*Pseudomonas fluorescens strain* M2	GU947867.1	97%
8	*Pseudomonas* sp. MFY222	AY331375.1	99%
9	*Pseudomonas* sp. R-45822	FR775122.1	100%
10	*Pseudomonas fluorescens*	EF408245.1	100%
11	*Pseudomonas* sp. CB10	EU482914.1	99%
12	*Pseudomonas* sp. MDT2-39-4	JX949559.1	99%
13	*Uncultured bacterium clone* 07MIC062	JF340783.1	99%
14	*Pseudomonas* sp. 01WB02.2-10	FM161381.1	95%
15	*Staphylococcus warneri strain* E3b	AY126243.1	98%
16	*Bacterium* het-w-28	KC810293.1	99%
17	*Pseudomonas* sp. CB10	EU482914.1	98%
18	*Pseudomonas* sp. IGS61	JN680232.1	99%
19	*Acinetobacter* sp. EAXY14	KC129073.1	99%
20	*Shewanella* sp. B157	FN295752.1	96%
21	*Microbulbifer maritimus strain* MTM147	HQ705770.1	93%
22	*Uncultured Escherichia* sp. N5	AM712054.1	98%
23	*Gamma proteobacterium* BAL382	KC140316.1	97%
24	*Alteromonadales bacterium* HD-I-03-6	AM931110.1	97%

**Table 2:** Component scores on volatile compounds of sturgeon inoculated different SSOs, respectively.

	PC1 (47.97%)	PC2 (35.41%)	PC3 (16.62%)
Control	−3.18	0.06	3.10
*Pseudomonas fluorescens*	5.61	−2.00	0.60
*Pseudomonas mandelii*	−2.93	−3.01	−2.42
*Shewanella putrefaciens*	0.50	4.92	−1.28

## Supplementary Materials

The following are available online at https://www.mdpi.com/article/10.3390/foods10092021/s1, Table S1: Volatile compounds identified in tray-packaging sturgeon fillets inoculated different SSOs, respectively, stored for 15 days on ice.
