# Supplementary material for: Identification of the Specific Spoilage Organism in Farmed Sturgeon (Acipenser baerii) Fillets and Its Associated Quality and Flavour Change during Ice Storage
Source: Foods. 2021 Aug 28;10(9):2021. doi: 10.3390/foods10092021 (PMC8469357; doi:10.3390/foods10092021)
Supplement: Supplementary file 1 [file foods-10-02021-s001.zip › foods-1319533-supplementary.pdf]

Supplementary Material

Identification of the specific spoilage organism in farmed sturgeon (*Acipenser baerii*) fillets and its associated quality and flavour change during ice storage

Table S1

Volatile compounds identified in tray-packaging sturgeon fillets inoculated different SSOs, respectively, stored for 15 d on ice

NO.	Volatile compound	RT /min	Relative content (%)			
			Control	*Pseudomonas fluorescens*	*Pseudomonas mandelii*	*Shewanella putrefaciens*
	**Aldehydes**					
1	Hexanal	5.73	2.62	0.90	2.60	0.79
2	Benzaldehyde	10.75	0.60	0.67	1.72	ND
3	Octanal	12.18	1.54	0.51	1.10	0.93
4	Nonanal	15.44	10.21	6.43	9.98	7.22
5	Decanal	18.52	6.71	1.93	5.18	4.52
6	Dodecanal	21.09	0.83	0.38	0.65	0.71
7	Tetradecanal	25.99	0.38	0.35	0.50	0.26
8	Hexadecanal	28.40	0.19	0.99	0.38	0.15
	**Ketones**					
9	1-(3,3-Dimethylbicyclo	19.48	ND	ND	ND	6.06

	[2.2.1]hept-2-yl)-ethanon					
10	2-Undecanone	20.76	0.26	1.54	0.54	0.34
11	6,10-dimethyl-5,9-Undecadien-2-one	23.64	1.30	0.90	0.78	0.96
	**Acids**					
12	Nonanoic acid	20.05	0.15	0.14	0.11	0.18
	**Esters**					
13	Ethyl hexanoate	12.04	1.23	ND	ND	0.53
	**Alcohols**					
14	2-Penten-1-ol	4.97	7.04	4.83	13.88	3.36
15	Hexanol	7.88	2.60	0.85	1.04	ND
16	1-Octen-3-ol	11.43	4.07	2.30	5.61	0.88
17	2-Ethyl-1-hexanol	13.02	35.53	46.01	30.74	33.27
18	2-hydroxycumene	14.80	0.90	1.12	0.90	1.06
	**Hydrocarbons**					
19	Azulene	17.86	3.83	5.00	3.97	3.68
20	Tetradecane	22.87	1.61	2.32	1.34	1.34
21	Acenaphthene	24.27	0.61	0.79	0.39	0.58
22	Pentadecane	24.45	1.21	1.74	1.45	1.84
23	Hexadecane	25.82	1.57	1.42	1.67	1.72
24	2,6,10,14-Tetramethyl-pentadecane	26.41	3.33	2.81	3.11	4.80
25	Heptadecane	27.06	2.67	2.22	1.74	3.98
26	Octadecane	28.20	0.79	0.42	1.07	1.48

27	2,6,10,14-Tetramethyl-hexadecane	28.28	0.36	0.18	0.59	0.70
28	2-Methyl-naphthalene	20.85	2.28	3.71	1.95	2.38
29	1-Methyl-naphthalene	21.18	0.96	1.60	0.69	1.01
30	1,3-Dimethyl-naphthalene	23.232	0.55	1.04	0.41	0.63
31	1,8-Dimethyl-naphthalene	23.31	0.28	0.44	0.26	0.28
	**Others**					
32	1,3-Dichloro-benzene	12.50	1.38	1.86	2.39	1.77
33	Benzothiazole	19.06	0.98	1.29	1.50	0.81
34	Butylated hydroxytoluene	24.50	1.43	1.86	1.76	11.78
35	Tetramethyl pyrazine	14.75	ND	1.45	ND	ND

ND: not detected; RT: retention time

The numbers of the volatile compounds in the table corresponded to the peak numbers in Figure 4(A、B、C、D).
